# Clindamycin and daptomycin failure in PVL‐positive MRSA infection of the skin and soft tissue

**DOI:** 10.1111/ddg.15988

**Published:** 2026-01-07

**Authors:** Michael Zolotas, Kamran Ghoreschi, Ulrike Blume‐Peytavi, Rasmus Leistner, Daniel Humme

**Affiliations:** ^1^ Department of Dermatology Venereology and Allergy Charité‐Universitätsmedizin Berlin Berlin Germany; ^2^ Institute of Hygiene and Environmental Medicine Charité‐Universitätsmedizin Berlin Berlin Germany

Dear Editors,

Panton‐Valentine‐leukocidin (PVL) is a potent pore‐forming toxin produced by certain strains of *Staphylococcus aureus* carrying the *lukS* and *lukF* genes. It is primarily associated with increased virulence and the development of severe skin and soft tissue infections.^1^, ^2^ The PVL‐toxin is known to enhance tissue necrosis as well as leukocytolysis and to provoke strong inflammatory responses, making the corresponding infections particularly challenging to manage.^1^, ^2^ Infection with PVL‐producing *Staphylococcus aureus* (PVL‐SA) can be confirmed with a PCR‐test for *lukS* and *lukF* genes.^3^ Recent studies have highlighted that PVL‐positive strains are more prevalent in complicated infections especially among vulnerable populations such as children. In the latter, apart from cutaneous, life‐threatening infections such as osteomyelitis, necrotizing pneumonia or fasciitis have also been described.^4^ Nevertheless, even young and healthy individuals may experience recurring severe infections due to PVL‐SA,^5^ often diagnosed with a delay of several months.^6^

Here we report the case of a 69‐years‐old male patient without any relevant comorbidities, especially no immunosuppression. The patient presented to our outpatient department with an erythematous, indurated subcutaneous tumor over his right shoulder measuring approximately 2 cm in diameter. His dermatologist had already performed an incision with drainage and initiated oral antibiotic treatment with clindamycin (600 mg three times daily) four days earlier. A further deep incision was performed under local anesthesia, yielding minimal pus for microbiological examination. At this point there was no leukocytosis and the CRP was only slightly elevated at 24.5 mg/L [norm: < 5 mg/L]. Continuation of the oral antibiotic therapy with clindamycin was recommended until antibiotic susceptibility results were available.

Three days later, the patient's condition had deteriorated, and he presented again with the subcutaneous tumor having grown to approximately 5 cm in diameter. Laboratory examination revealed an elevated CRP at 90 mg/L and leukocytosis of 13.6/nL [norm: 3.9–10.5/nL] with neutrophilia at 10.8/nl [norm: 1.5–7.7/nL]. Culture results were positive for methicillin‐resistant *Staphylococcus aureus* (MRSA) with resistance to clindamycin. This necessitated inpatient admission on the same day and initiation of intravenous vancomycin therapy (1.5 g twice daily) according to the antibiotic susceptibility testing (online supplementary Table S1), along with decolonization measures. A sonographic examination of the right shoulder revealed an approximately 2 cm deep lesion with heterogeneous echogenicity, but no clear evidence of an abscess cavity. After two days, due to repeatedly insufficient vancomycin serum concentration as well as further worsening of the skin lesion (Figure 1a) and inflammatory parameters (CRP: 190 mg/L, leukocytes: 11.4/nL), the antibiotic therapy was switched to daptomycin (10 mg/kg once daily) in consistency with the antibiotic susceptibility testing.

**FIGURE 1:**
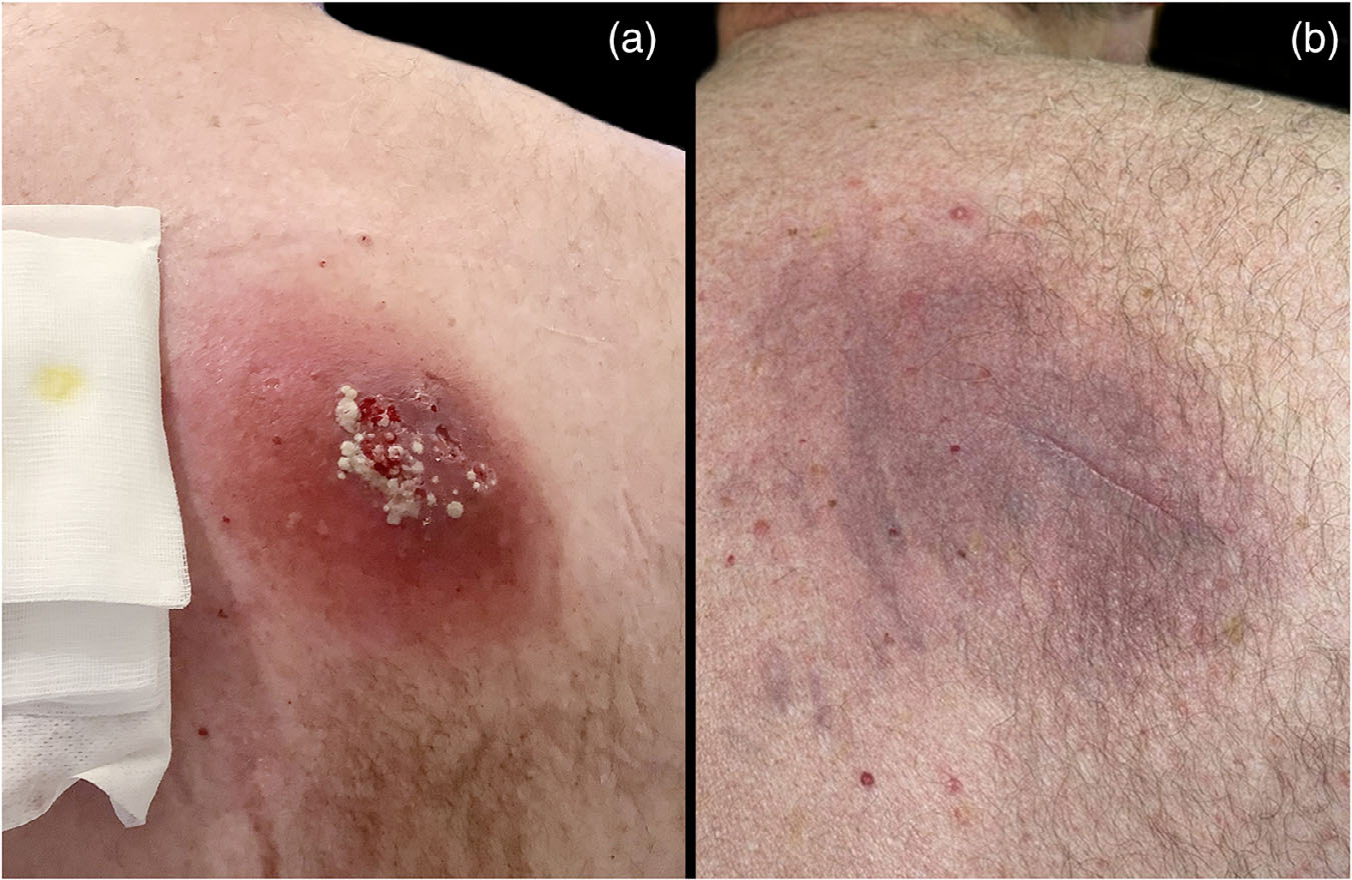
Photodocumentation of the patient: a) two days after inpatient admission; b) twenty days after discharge.

Subsequent deep incision and drainage of the wound on the following day revealed slight improvement under daptomycin, although the purulent secretion persisted. An MRI‐scan performed a few days later showed an inflammatory mass of approximately 10 cm in diameter and 2.5 cm in depth with significant surrounding edema affecting the fascia of the trapezius muscle, but no intramuscular involvement (Figure 2). Despite ongoing treatment, the infection parameters remained elevated for the next eighteen days and cultures from the wound confirmed persistent MRSA. A further MRI‐scan showed only minor regression of the lesion.

**FIGURE 2:**
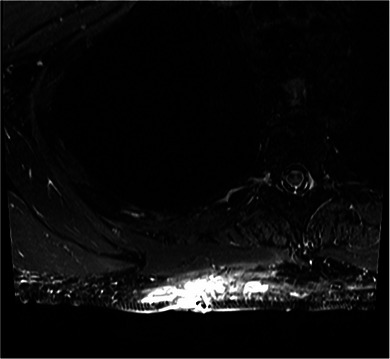
Exemplary image from the MRI‐scan performed nine days after inpatient admission

After three weeks of daptomycin therapy, microbiologic evaluation still demonstrated MRSA growth in the cutaneous tumor, and PCR additionally confirmed PVL‐production. Upon consulting infectious disease specialists, this prompted a further change in antimicrobial regimen to intravenous administration of co‐trimoxazole (960 mg) and rifampicin (450 mg) twice daily based on the antibiotic susceptibility testing. This new therapeutic regimen finally led to marked clinical improvement enabling discharge of the patient nine days later with subsequent oral treatment consisting of a co‐trimoxazole monotherapy for a further week. Follow‐up cultures were negative, as were subsequent nasal, throat and wound samples taken in the following weeks. Ultimately, the wound healed completely (Figure 1b) and no relapse was recorded thereafter.

This case of a severe, complicated skin and soft tissue infection highlights the significant therapeutic challenges associated with PVL‐MRSA – a virulent and increasingly prevalent pathogen.^7^ Treatment was particularly difficult due to resistance against clindamycin and an unexpected clinical failure of daptomycin, despite in vitro susceptibility. Clindamycin resistance in MRSA is not uncommon and may also occur via inducible mechanisms, leading to clinical failure if undetected.^8^ In contrast, daptomycin non‐susceptibility of MRSA is rare,^9^ and in combination with PVL production, it markedly enhances the pathogen's virulence.

The clinical course, which involved hospitalization for over four weeks, underscores the importance of early and accurate diagnosis with ongoing microbiological surveillance, including appropriate cultures and susceptibility testing in all severe skin and soft tissue infections, not only at the onset but also during therapy if symptoms persist. Furthermore, it reflects the broader and escalating threat of antimicrobial resistance,^10^ reinforcing the urgent need for tailored antimicrobial strategies and adherence to standardized treatment protocols for resistant pathogens.

## CONFLICT OF INTEREST STATEMENT

The authors report no conflicts of interest

Open access funding enabled and organized by Projekt DEAL.

## Supporting information



Supporting Information
