# Supplementary material for: Clindamycin and daptomycin failure in PVL‐positive MRSA infection of the skin and soft tissue
Source: J Dtsch Dermatol Ges. 2026 Jan 7;24(5):674–6. doi: 10.1111/ddg.15988 (PMC13140117; doi:10.1111/ddg.15988)
Supplement: Supplementary file 1 — Supporting Information [file DDG-24-674-s001.docx]

**SUPPLEMENTARY TABLE 1**

| Antibiotic | Result | MIC |
| --- | --- | --- |
| Penicillin | R | ≥ 0.5 |
| Ampicillin | R |  |
| Oxacillin | R | ≥ 4.0 |
| Ampicillin/Sulbactam | R |  |
| Piperacillin | R |  |
| Piperacillin/Tazobactam | R |  |
| Cefazolin | R |  |
| Cefuroxime | R |  |
| Ceftriaxone | R |  |
| Imipenem | R |  |
| Meropenem | R |  |
| Levofloxacin | I | 0.25 |
| Gentamicin | S | ≤ 0.5 |
| Tobramycin | S |  |
| Teicoplanin | S | ≤ 0.5 |
| Vancomycin | S | 1.0 |
| Erythromycin | R | ≥ 8.0 |
| Clindamycin | R | ≥ 4.0 |
| Doxycycline | R | ≥ 16.0 |
| Tigecycline | S | ≤ 0.12 |
| Linezolid | S | 1.0 |
| Daptomycin | S | 0.25 |
| Fosfomycin | S | ≤ 8.0 |
| Fusidic acid | S | ≤ 0.5 |
| Mupirocin | S | ≤ 1.0 |
| Rifampicin | S | ≤ 0.03 |
| Trimethoprim/Sulfamethoxazole  (Co-trimoxazole) | S | ≤ 10.0 |

Supplementary Table 1: Resistogram of the PVL-MRSA detected in the sample from January 19, 2024 (R: resistant; I: intermediate, i.e. susceptible under increased exposure; S: susceptible under standard dosing regimen; MIC: minimum inhibitory concentration in mg/l – interpretation according to EUCAST-Standard 13.0)
